
JOURNAL INFORMATION
==============================
NLM Title Abbreviation: Open Forum Infect Dis
Journal ID: Open Forum Infect Dis
NLM Journal ID: 101637045
Journal ID: ofid

Open Forum Infectious Diseases

EISSN: 2328-8957
Publisher: Oxford University Press

ARTICLE INFORMATION
==============================
PMCID: PMC10036674
PMID: 36968968
DOI: 10.1093/ofid/ofad113
Article ID: ofad113
Article version: 1
Subjects: Insight Article, AcademicSubjects/MED00290

Effect of fecal microbial transplantation on Clostridioides difficile infection: dysbiosis, metabolites and health related quality of life

Garey Kevin W Department of Pharmacy University of Houston

Feuerstadt Paul PACT Gastrointestinal Associates & Yale University

Dubberke Erik R Division of Infectious Diseases, U Washington & Barns Jewish Hospital, St Louis

Guo Amy Ferring Pharmaceuticals

Tillotson Glenn S GST Micro LLC

Corresponding author name: Glenn Tillotsongtillotson@gstmicro.comAlternate author: Paul Feuerstadtpfeuerstadt@gastrocenter.org
Publication date: 2023 Mar
Electronic publication date: 2023 Mar 24
Volume: 10
Issue: 3
Electronic Location ID: ofad113
2023 Mar 24
Copyright: © The Author(s) 2023. Published by Oxford University Press for the Infectious Diseases Society of America.
Copyright year: 2023
License: This is an Open Access article distributed under the terms of the Creative Commons Attribution License (https://creativecommons.org/licenses/by/4.0/), which permits unrestricted reuse, distribution, and reproduction in any medium, provided the original work is properly cited.
License URL: https://creativecommons.org/licenses/by/4.0/

Page count: 5

==============================

Open Forum Infectious Diseases

Open Forum Infectious Diseases 2023; 10: 3
doi: 10.1093/ofid/ofad113

InSight

### Effect of fecal microbial transplantation on *Clostridioides difficile* infection: dysbiosis, metabolites and health related quality of life

Kevin W Garey [1], Paul Feuerstadt [2], Erik R Dubberke [3], Amy Guo [4], Glenn S Tillotson [5]

[1] Department of Pharmacy University of Houston
[2] PACT Gastrointestinal Associates & Yale University
[3] Division of Infectious Diseases, U Washington & Barns Jewish Hospital, St Louis
[4] Ferring Pharmaceuticals
[5] GST Micro LLC

### The gut-brain axis

A complex bidirectional communication system exists between the gastrointestinal tract and the brain, called the "gut-brain axis" [1]. The growing recognition of the microbiome's importance in modulating health has prompted an extension of this term to the "microbiota-gut-brain axis", which represents a complex network of communication between the gut, microbiota, and the brain that modulates immune, gastrointestinal, and central nervous system (CNS) functions [1-3]. Mechanisms of communication include: (1) neurotransmitters; (2) microbial byproducts or metabolites including short-chain fatty acids (SCFA) and bile acids; (3) cytokines and immune cell activation; (4) neural networks including enteric nervous system (ENS) and vagal nerve activation; (5) and hypothalamic-pituitary-adrenal (HPA) axis modulation [4-8].

Central to the discussion herein is the integral role of both neurotransmitters and microbial metabolites within the microbiota-gut-brain axis:

- The gut microbiota can produce key central neurotransmitters such as gamma-aminobutyric acid (GABA), norepinephrine, dopamine, and serotonin, which have important influences that extend beyond the gut to the brain [9]. Accumulating evidence suggests that manipulation of neurotransmitters by bacteria may have important physiological implications [9-11]. Notably, modulation of glutamate and GABA, which are the major excitatory and inhibitory neurotransmitters found in the CNS, respectively [12, 13], may play a role in the development of various mood, cognitive, and behavioral functions [11, 14-17].
- The metabolome, which can be defined as the complete complement of all small molecule metabolites [18], is another way the microbiome plays a central role in gut-brain communication. This occurs via a number of different metabolites including SCFAs, bile acids, and tryptophan, which can activate several mechanisms in the brain.

### Dysbiosis in *Clostridioides difficile* infection

*Clostridioides difficile* infection (CDI), the most common cause of antibiotic- and healthcare-associated infective diarrhea in the US [19, 20] characterized by a disrupted microbiome [21-23], has health-related quality of life (HRQOL) implications [24-31]. Most *Clostridioides difficile* infection patients admit their daily activities are impacted [29, 32, 33] and the psychological and emotional impact is high; patients describe a wide array of emotions that they have experienced throughout their infection, including:

- Feelings of shame, humiliation, embarrassment, stress, depression, anxiety, and frustration [28, 31-37].
- Fear or worry related to worsening CDI, getting sick again, having uncontrolled symptoms for a longer period of time, never being cured, or infecting others [29, 31, 33, 34, 37].
- Concern about hospitalization due to a lack of trust in the ability of hospitals to adequately control and manage infections [34].
- Feelings of loneliness and worry when hospitalized [31]

Patients often intentionally take steps to evade certain social situations; this can have a profound impact on social lives as it can lead to the avoidance of venues or events outside of the home, including restaurants, public bathrooms, vacations, weddings, funerals, and more [26, 29, 31, 33, 34, 37, 38].

© The Author(s) 2023. Published by Oxford University Press for the Infectious Diseases Society of America. This is an Open Access article distributed under the terms of the Creative Commons Attribution License (https://creativecommons.org/licenses/by/4.0/), which permits unrestricted reuse, distribution, and reproduction in any medium, provided the original work is properly cited.

Microbiota-gut-brain axis communication mechanisms
Blood-brain barrier
Brain
Blood
Key central neurotransmitters produced by gut microbiota can have important influences beyond the gut
• GABA
• Norepinephrine
• Dopamine
• Serotonin
• Histamine
Cannot cross the blood brain barrier and act indirectly through the vagal nerve to modulate brain function
1
Neurotransmitters
2
Microbial metabolites
SCFAs (e.g., butyrate, proprionate, acetate)
Largely produced in the colon by Bacteroides, Bifodobacterium, Eubacterium, Faecalibacterium, Lactobacillus, Lachnospiraceae, Blautia, Coprococcus, Propionibacterium, Roseburia and Veilonella
• Modulate neurotrophic factors, neurotransmitters, neurogenesis
• Reduce neuroinflammation
• Induce production of several hormones and neurotransmitters (e.g., GABA, serotonin)
Bile acids (e.g., deoxycholic, lithocholic acids)
Secondary bile acids are generated from Clostridium, Enterococcus, Bifidobacterium, and Lactobacillus, which exhibit bile salt hydroxylase activity
• Directly regulate brain function by local modulation of receptors
• Key regulators of gut microbiota composition
• Gut microbiota is a major mediator of bile acid chemistry (composition of bile acid pool is mediated by bacterial metabolism in intestinal tract)
Tryptophan
Mainly derived from the diet and metabolized by Firmicutes, Bacteroidetes, Actinobacteria, Proteobacteria, and Fusobacteria to various products including serotonin, kynurenines, tryptamine, and indolic compounds
• Neuroendocrine and immune-modulatory actions
Cross the blood-brain barrier and activate several mechanisms
Gut epithelium
GUT MICROBIOTA
Healthy gut microbiota:
Firmicutes and Bacteroidetes constitute the vast majority of the dominant human gut microbiota, followed by Actinobacteria and Proteobacteria

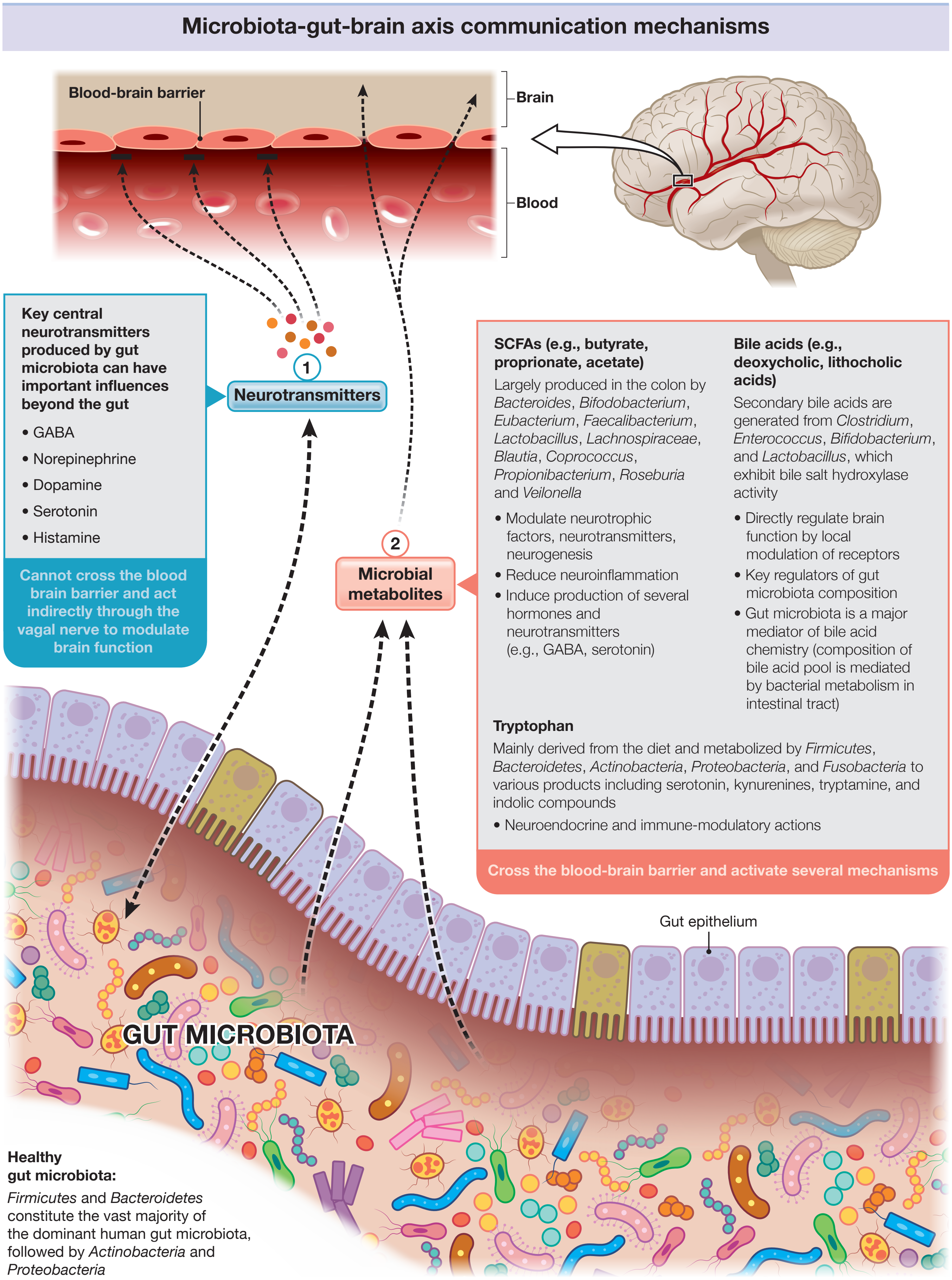

### REBYOTA™ (fecal microbiota, live-jslm; FMBL) has been successful in the treatment of recurrent CDI

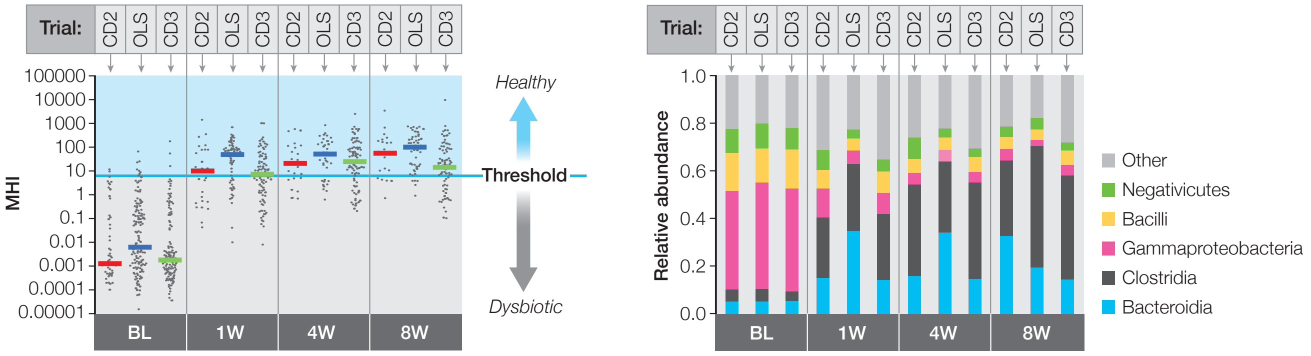

### Bile acids are rapidly restored to healthier compositions after FMBL treatment with a significant secondary to primary bile acid (S:P) ratio increase relative to non-responders

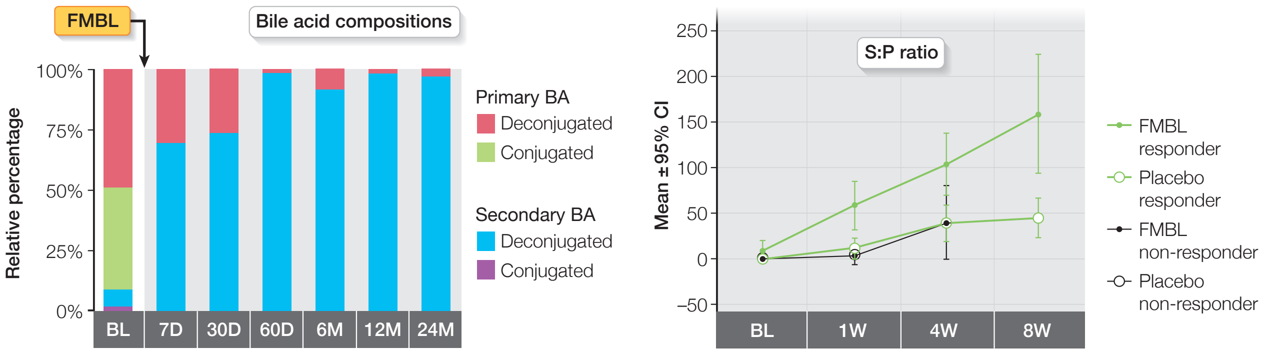

### FMBL treatment leads to sustained improvements in Cdiff32 with statistically significant differences in the adjusted mental domain and total score

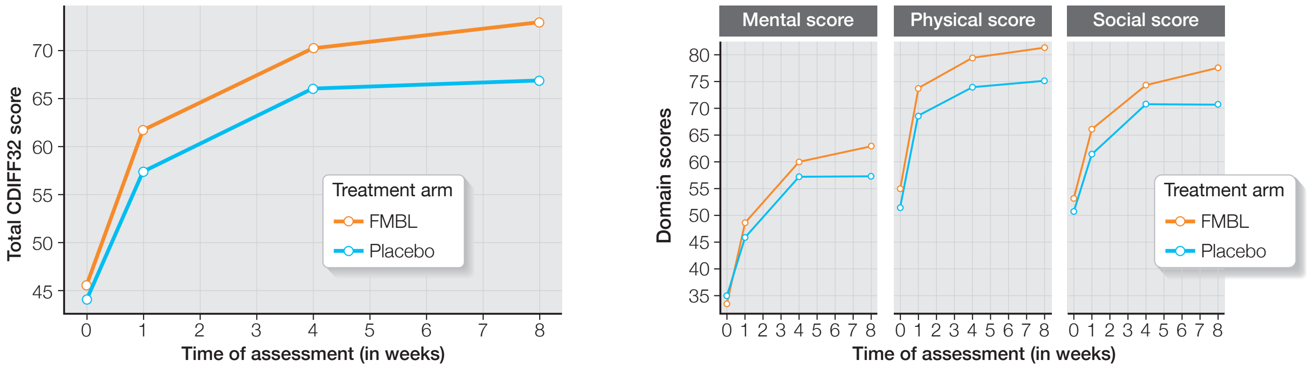

**Legend:**

**CD2** = comparative phase II, **OLS** = open label study, **CD3** = comparative phase III study, **BL** = baseline, **1W** = 1 week, **4W** = 4 weeks, **8W** = 8 weeks

### HRQOL assessments

Several instruments are used to assess HRQOL in the clinical setting, including the widely employed 36-item Short Form 36 (SF-36) Health Survey [39] and the EQ-5D [40]; however, there was a need to develop and validate a disease-specific instrument to assess HRQOL changes related to CDI with a focus on recurrent CDI. As such, the Cdiff32, a disease-specific scale that contains 32 items related to the physical, mental, and social health in patients with CDI, was introduced in 2016 [41]. This standardized tool has since been utilized in clinical trials [41] and is the HRQOL instrument to use in patients with CDI.

### Fecal microbial transplantation

Fecal microbial transplantation (FMT) has been proposed to improve neurological and psychological symptoms in CDI by likely modulating the gut-brain axis [42, 43]. Its effectiveness is largely due to the following:

- Direct microbiological mechanisms: successful FMT administration is marked by an eradication of key Proteobacteria species and a corresponding restoration of key Bacteroidetes and Firmicutes species, helping to prevent colonization and/or outgrowth of C. difficile [21, 44]
- Gut microbial bile acid metabolism: FMT restores the capacity of the microbial community to convert primary bile acids (which stimulate spore germination) into secondary bile acids (which inhibit C. difficile germination and epithelial apoptosis) [45-47]
- Repopulation of SCFA bacteria: FMT leads to sustained increases in several butyrate producers, including members of the Clostridiales clade [47]

### REBYOTA$^{TM}$ (fecal microbiota, live-jslm; FMBL) improves HRQOL

REBYOTA$^{TM}$ (fecal microbiota, live-jslm; FMBL) (Rebiotix Inc., Roseville, MN, a Ferring company) is a recently FDA approved live biotherapeutic containing Bacteroidetes and other key species that has demonstrated safety and efficacy in adults with rCDI [48-50]. In the 6-month pivotal PUNCH CD3 clinical trial, FMBL-treated patients had more profound and sustained improvements at week eight compared to placebo with statistically significant differences in the adjusted mental domain and total score [51]. Similar results were found among FMBL responders versus placebo responders at week eight for the mental domain of Cdiff32; even among the few clinically non-responders, there were numerical improvements in all domains of Cdiff32 for FMBL-treated patients but not placebo-treated patients [52].

### Conclusion

- Neurotransmitters and microbial metabolites play important roles within the microbiota-gut-brain axis
- A disrupted microbiome along with associated alterations in microbial metabolites has been implicated in CDI
- While CDI is characterized by significant diarrheal disease, it has underappreciated HRQOL implications
- FMBL promotes a more diverse and balanced microbiota composition that has direct antimicrobial actions
- A healthier microbiome increases SCFA production and promotes conversion of primary to secondary bile acids
- FMBL improves various aspects of HRQOL, which can be seen as early as one-week post-treatment

| | |
|---|---|
| Corresponding author name: | **Glenn Tillotson** – gtillotson@gstmicro.com *alternate author*: **Paul Feuerstadt** – pfeuerstadt@gastrocenter.org |
| | |
|---|---|
| InSight title: | **Effect of fecal microbial transplantation on *Clostridioides difficile* infection: dysbiosis, metabolites and health related quality of life** |
| Sponsor: | This InSight is sponsored by **Ferring Pharmaceuticals.** |
| DOI: | https://10.1093/ofid/ofad113 |

OXFORD UNIVERSITY PRESS

Open Forum Infectious Diseases

© The Author(s) 2023. Published by Oxford University Press for the Infectious Diseases Society of America. This is an Open Access article distributed under the terms of the Creative Commons Attribution License (https://creativecommons.org/licenses/by/4.0/), which permits unrestricted reuse, distribution, and reproduction in any medium, provided the original work is properly cited.

### References

1. Rhee, S.H., C. Pothoulakis, and E.A. Mayer, *Principles and clinical implications of the brain-gut-enteric microbiota axis*. Nat Rev Gastroenterol Hepatol, 2009. **6**(5): p. 306-14.
2. Collins, S.M., M. Surette, and P. Bercik, *The interplay between the intestinal microbiota and the brain*. Nat Rev Microbiol, 2012. **10**(11): p. 735-42.
3. Carabotti, M., et al., *The gut-brain axis: interactions between enteric microbiota, central and enteric nervous systems*. Ann Gastroenterol, 2015. **28**(2): p. 203-209.
4. Rea, K., T.G. Dinan, and J.F. Cryan, *Gut Microbiota: A Perspective for Psychiatrists*. Neuropsychobiology, 2020. **79**(1): p. 50-62.
5. O'Riordan, K.J., et al., *Short chain fatty acids: Microbial metabolites for gut-brain axis signalling*. Mol Cell Endocrinol, 2022. **546**: p. 111572.

6. Margolis, K.G., J.F. Cryan, and E.A. Mayer, *The Microbiota-Gut-Brain Axis: From Motility to Mood*. Gastroenterology, 2021. **160**(5): p. 1486-1501.
7. Gubert, C., J. Gasparotto, and H.M. L, *Convergent pathways of the gut microbiota-brain axis and neurodegenerative disorders*. Gastroenterol Rep Oxf), 2022. **10**: p. goac017.
8. Morais, L.H., H.L.t. Schreiber, and S.K. Mazmanian, *The gut microbiota-brain axis in behaviour and brain disorders*. Nat Rev Microbiol, 2021. **9**(4): p. 241-255.
9. Strandwitz, P., *Neurotransmitter modulation by the gut microbiota*. Brain Res, 2018. **1693**(Pt B): p. 128-133.
10. Huang, F. and X. Wu, *Brain Neurotransmitter Modulation by Gut Microbiota in Anxiety and Depression*. Front Cell Dev Biol, 2021. **9**: p. 649103.
11. Ortega, M.A., et al., *Gut Microbiota Metabolites in Major Depressive Disorder-Deep Insights into Their Pathophysiological Role and Potential Translational Applications*. Metabolites, 2022. **12**(1).
12. Hyland, N.P. and J.F. Cryan, *A Gut Feeling about GABA: Focus on GABA(B) Receptors*. Front Pharmacol, 2010. **1**: p. 124.
13. Julio-Pieper, M., et al., *Regulation of the brain-gut axis by group III metabotropic glutamate receptors*. Eur J Pharmacol, 2013. **698**(1-3): p. 19-30.
14. Filpa, V., et al., *Role of glutamatergic neurotransmission in the enteric nervous system and brain-gut axis in health and disease*. Neuropharmacology, 2016. **111**: p. 14-33.
15. Baj, A., et al., *Glutamatergic Signaling Along The Microbiota-Gut-Brain Axis*. Int J Mol Sci, 2019. **20**(6).
16. Femenia, T., et al., *Dysfunctional hippocampal activity affects emotion and cognition in mood disorders*. Brain Res, 2012. **1476**: p. 58-70.
17. Dicks, L.M.T., *Gut Bacteria and Neurotransmitters*. Microorganisms, 2022. **10**(9).
18. Wishart, D.S., et al., HMDB: *the Human Metabolome Database*. Nucleic Acids Res, 2007. **35**(Database issue): p. D521-6.
19. Lessa, F.C., et al., *Burden of Clostridium difficile infection in the United States*. N Engl J Med, 2015. **372**(9): p. 825-34.
20. Magill, S.S., et al., *Changes in Prevalence of Health Care-Associated Infections in U.S. Hospitals*. N Engl J Med, 2018. **379**(18): p. 1732-1744.
21. Shahinas, D., et al., *Toward an understanding of changes in diversity associated with fecal microbiome transplantation based on 16S rRNA gene deep sequencing*. mBio, 2012. **3**(5).
22. Chang, J.Y., et al., *Decreased diversity of the fecal Microbiome in recurrent Clostridium difficile-associated diarrhea*. J Infect Dis, 2008. **197**(3): p. 435-8.
23. Seekatz, A.M., et al., *Dynamics of the fecal microbiome in patients with recurrent and nonrecurrent Clostridium difficile infection*. Genome Med, 2016. **8**(1): p. 47.
24. Hengel, R.L., et al., *Recurrent Clostridioides difficile infection worsens anxiety-related patient-reported quality of life*. J Patient Rep Outcomes, 2022. **6**(1): p. 49.
25. Han, Z., et al., *Impact of Clostridioides difficile infection on patient-reported quality of life*. Infect Control Hosp Epidemiol, 2021: p. 1-6.
26. Heinrich, K., et al., *Impaired Quality of Life, Work, and Activities Among Adults with Clostridium difficile Infection: A Multinational Survey*. Dig Dis Sci, 2018. **63**(11): p. 2864-2873.
27. Wilcox, M.H., et al., *Impact of recurrent Clostridium difficile infection: hospitalization and patient quality of life*. J Antimicrob Chemother, 2017. **72**(9): p. 2647-2656.
28. Barbut, F., et al., *Quality of life and utility decrement associated with Clostridium difficile infection in a French hospital setting. Health Qual Life Outcomes*, 2019. **17**(1): p. 6.
29. Lurienne, L., et al., *Perception of quality of life in people experiencing or having experienced a Clostridioides difficile infection: a US population survey*. J Patient Rep Outcomes, 2020. **4**(1): p. 14.
30. Vent-Schmidt, J., et al., *Patient Experiences with Clostridioides difficile Infection: Results of a Canada-Wide Survey*. Patient Prefer Adherence, 2020. **14**: p. 33-43.
31. Guillemin, I., et al., *Patients' experience and perception of hospital-treated Clostridium difficile infections: a qualitative study*. Patient, 2014. **7**(1): p. 97-105.
32. Pakyz, A.L., et al., *Fecal microbiota transplantation for recurrent Clostridium difficile infection: The patient experience*. Am J Infect Control, 2016. **44**(5): p. 554-9.
33. Weaver, F.M., et al., *The Impact of Recurrent Clostridium difficile Infection on Patients' Prevention Behaviors*. Infect Control Hosp Epidemiol, 2017. **38**(11): p. 1351-1357.
34. Downie, K., M.J. Salpeter, and S. Smita Hota, *Exploring the patient experience with recurrent Clostridium difficile infection in Ontario, Canada*. Canadian Journal of Infection Control, 2017. **32**(2): p. 81-86.
35. Rogers, M.A., et al., *Depression, antidepressant medications, and risk of Clostridium difficile infection*. BMC Med, 2013. **11**: p. 121.
36. Mikocka-Walus, A., *Depression and use of antidepressants is associated with increased risk of Clostridium difficile infection*. Evid Based Ment Health, 2013. **16**(4): p. 95.
37. Zellmer, C., et al., *Patient Perspectives on Fecal Microbiota Transplantation for Clostridium Difficile Infection*. Infect Dis Ther, 2016. **5**(2): p. 155-64.
38. Donskey, C.J., *Clostridium difficile in Older Adults*. Infect Dis Clin North Am, 2017. **31**(4): p. 743-756.
39. Ware, J.E., Jr. and C.D. Sherbourne, *The MOS 36-item short-form health survey (SF-36). I. Conceptual framework and item selection*. Med Care, 1992. **30**(6): p. 473-83.
40. Kleinman, L., et al., The CDI-DaySyms: *Content Development of a New Patient-Reported Outcome Questionnaire for Symptoms of Clostridium difficile Infection*. Value Health, 2018. **21**(4): p. 441-448.
41. Garey, K.W., et al., *Development and Validation of a Clostridium difficile Health-related Quality-of-Life Questionnaire*. J Clin Gastroenterol, 2016. **50**(8): p. 631-7.
42. Xu, H.M., et al., *Fecal Microbiota Transplantation: A New Therapeutic Attempt from the Gut to the Brain*. Gastroenterol Res Pract, 2021. **2021**: p. 6699268.
43. Vendrik, K.E.W., et al., *Fecal Microbiota Transplantation in Neurological Disorders*. Front Cell Infect Microbiol, 2020. **10**: p. 98.
44. Soveral, L.F., et al., *Immunological mechanisms of fecal microbiota transplantation in recurrent Clostridioides difficile infection*. World J Gastroenterol, 2022. **28**(33): p. 4762-4772.
45. Weingarden, A.R., et al., *Microbiota transplantation restores normal fecal bile acid composition in recurrent Clostridium difficile infection*. Am J Physiol Gastrointest Liver Physiol, 2014. **306**(4): p. G310-9.
46. Weingarden, A.R., et al., *Changes in Colonic Bile Acid Composition following Fecal Microbiota Transplantation Are Sufficient to Control Clostridium difficile Germination and Growth*. PLoS One, 2016. **11**(1): p. e0147210.
47. Seekatz, A.M., et al., *Restoration of short chain fatty acid and bile acid metabolism following fecal microbiota transplantation in patients with recurrent Clostridium difficile infection*. Anaerobe, 2018. **53**: p. 64-73.
48. Dubberke, E., et al., *Final results from a phase II randomized, placebo-controlled clinical trial of RBX2660: a microbiota-based drug for the prevention of recurrent Clostridioides difficile infection* 2022.
49. Orenstein, R., et al., *Durable reduction of Clostridioides difficile infection recurrence and microbiome restoration after treatment with RBX2660: results from an open-label phase 2 clinical trial*. BMC Infect Dis, 2022. **22**(1): p. 245.
50. Khanna, S., et al., *Efficacy and safety of RBX2660 in reducing recurrent Clostridioides difficile infection in patients with underlying gastrointestinal comorbidities [poster], in In: ACG; October 21-26*. 2022: Charlotte, North Carolina.
51. Feuerstadt, P., et al., *Significant improvement in health-related quality of life (HRQL) with RBX2660: results from a phase 3 randomized, placebo-controlled trial in recurrent Clostridioides difficile infection (PUNCH CD3) [poster], in In: ID Week; October 19-23*. 2022: Washington, District of Columbia.
52. Feuerstadt, P., et al., *Health-related quality of life of week 8 responders and non-responders: results from the RBX2660 phase 3 randomized, placebo-controlled trial in recurrent clostridioides difficile infection (PUNCH CD3) [abstract], in In: ACG; October 21-26*. 2022: Charlotte, North Carolina.

**Conflict of Interest Statement:**

**KG**: an employee of University of Houston College of Pharmacy, consultant for Ferring Pharmaceuticals, received research grant from Acurx, Summit, Paratek Pharmaceuticals and Seres Therapeutics. **PF**: an employee of Yale University School of Medicine, consultant, speaker's bureau and advisory board for Ferring/Rebiotix, consultant and advisory board for Seres Therapeutics, consultant for Merck and Co. and advisory board for Takeda Pharmaceuticals. **ED**: an employee of Washington University School of Medicine, advisor/consultant for Abbott, Ferring Pharmaceuticals, Merck, Pfizer, Seres Therapeutics, Summit, received grant/research support from Ferring Pharmaceuticals, Pfizer and Synthetic Biologics. **AG**: an employee of Ferring Pharmaceuticals. **GT**: an employee of GST Micro LLC, consultant to Ferring Pharmaceuticals, Spero Therapeutics and Paratek Pharmaceuticals.
